# Effects of seaweed fertilizer application on crops’ yield and quality in field conditions in China-A meta-analysis

**DOI:** 10.1371/journal.pone.0307517

**Published:** 2024-07-18

**Authors:** Baolei Pei, Yunpeng Zhang, Ting Liu, Jian Cao, Huai Ji, Zhenzhu Hu, Xinxin Wu, Feibing Wang, Ying Lu, Ningyi Chen, Junkang Zhou, Boqing Chen, Sa Zhou

**Affiliations:** 1 Jiangsu Provincial Agricultural Green and Low Carbon Production Technology Engineering Research Center, College of Life Science and Food Engineering, Huaiyin Institute of Technology, Huai’an, Jiangsu, China; 2 Key Laboratory of Silviculture and Conservation of the Ministry of Education, National Energy R&D Center for Non-food Biomass, College of Forestry, Beijing Forestry University, Beijing, China; 3 College of Forestry, Nanjing Forestry University, Nanjing, Jiangsu, China; 4 Key Laboratory of Industrial Fermentation Microbiology of the Ministry of Education, College of Biotechnology, Tianjin University of Science and Technology, Tianjin, China; University of the West Indies, TRINIDAD AND TOBAGO

## Abstract

Seaweed fertilizer, formulated primarily with seaweed extract as its main ingredient, has been extensively studied and found to significantly improve nutrient use efficiency, increase crop yield and quality, and enhance soil properties under field conditions. This growing body of evidence shows that seaweed fertilizer is a suitable option for sustainable agriculture in China. However, a comprehensive and quantitative analysis of the overall effects of seaweed fertilizer application in China is lacking. To address this gap, we conducted a meta-analysis of relevant studies on the effects of seaweed fertilizers under field conditions in China with MetaWin and SPSS software. Our analysis examined the effects of seaweed fertilizers on crop yield, quality, and growth under different preparation methods, application techniques, and regions. Our results showed that the application of seaweed fertilizer led to a significant average increase in crop yield of 15.17% compared with the control treatments. Root & tuber crops exhibited the most pronounced response, with a yield boost of 21.19%. Moreover, seaweed fertilizer application significantly improved crop quality, with elevations in the sugar-acid ratio (38.32%) vitamin C (18.07%), starch (19.65%), and protein (11.45%). In addition, plant growth parameters such as height, stem thickness, root weight, and leaf area showed significant enhancement with seaweed fertilizer use. The yield-increasing effect of seaweed fertilizers varied depending on their preparation and use method, climate, and soil of application location. Our study provides fundamental reference data for the efficient and scientific application of seaweed fertilizers in agricultural practices.

## Introduction

With over 18% of the global population residing on less than 7% of the world’s farmland in China, the country faces significant challenges in food production due to the intensive use of farmland [1,2]. Over the last few decades, excessive and improper application of chemical fertilizers has led to low nutrient use efficiency, water and air pollution, and soil degradation [3–5]. In response to these issues and to promote the sustainability of agriculture, China has implemented various policies, including the ’Zero Increase Action Plan’ for chemical fertilizers and the expansion of functional fertilizers use [6].

Seaweed fertilizer, prepared based on seaweed extracts, has been found to confer various benefits to plants. i) It provides plants with unique nutrients, including rich macro and secondary macro elements (such as N, P, K, Ca, Mg) as well as diverse forms of trace and rare elements (such as zinc, bromine, and iodine), which are inherent to marine organisms [7–10]. ii) Furthermore, it stimulates plant growth and defense mechanisms by providing plant growth regulators like auxins, cytokinins, and gibberellins. These compounds promote root development, enhance flowering, and bolster overall plant vigor [11–13]. Additionally, these growth regulators aid in stress tolerance, enabling plants to withstand adverse environmental conditions such as drought, salinity, and temperature fluctuations [14–16]. iii) improving the soil condition. The application of seaweed fertilizer has been found to improve soil conditions by increasing microbial activity and nutrient cycling. Seaweed extracts serve as natural soil conditioners, improving soil structure and water-holding capacity [13,17]. They foster beneficial microbial populations, crucial for nutrient availability and disease suppression [18–20]. Through a specific extraction process, active compounds are derived from seaweed, which can be utilized either alone as a ’biostimulant’ at recommended rates as low as 0.5 kg/ha or 0.5 L/ha through foliar application or fertigation. Alternatively, they can be incorporated as additive agents for organic or chemical materials to produce seaweed organic or chemical fertilizers. Although seaweed fertilizers tend to be more costly compared to those lacking seaweed extracts, it’s market is poised for substantial growth. According to The World Bank report, due to the seaweed fertilizer’s multiple functions and high profit, its market will reach 1.8 billion in 2030 from 1 billion in 2023 [21].

However, the efficacy of seaweed fertilizer described above is primarily contingent upon the inherent physicochemical properties of the original seaweed species [7,22,23]. Moreover, variations in seaweed fertilizer production processes, application methods, geographical locations, soil types, crop varieties, etc., further influence its effects. In a meta-analysis, researchers found the disparate effects of seaweed extract on root-knot nematodes, attributing the variations to differences in plant species, seaweed species, and nematode strains [24]. However, a comprehensive statistical elucidation of the systemic effects of seaweed fertilizer on crop yield and quality, along with the factors modulating these effects, is yet to be undertaken. In addition, the diversity of climate, soil, crops, and seaweed fertilizer products are all relatively high in China. Thus, we conducted a meta-analysis of the effects of seaweed fertilizer and their influence factors in field conditions in China. The statistical data in this study could be an appropriate reference for evaluating the potential of seaweed fertilizer in practical field farming.

## Materials and methods

### Studies identification

**Fig 1:**
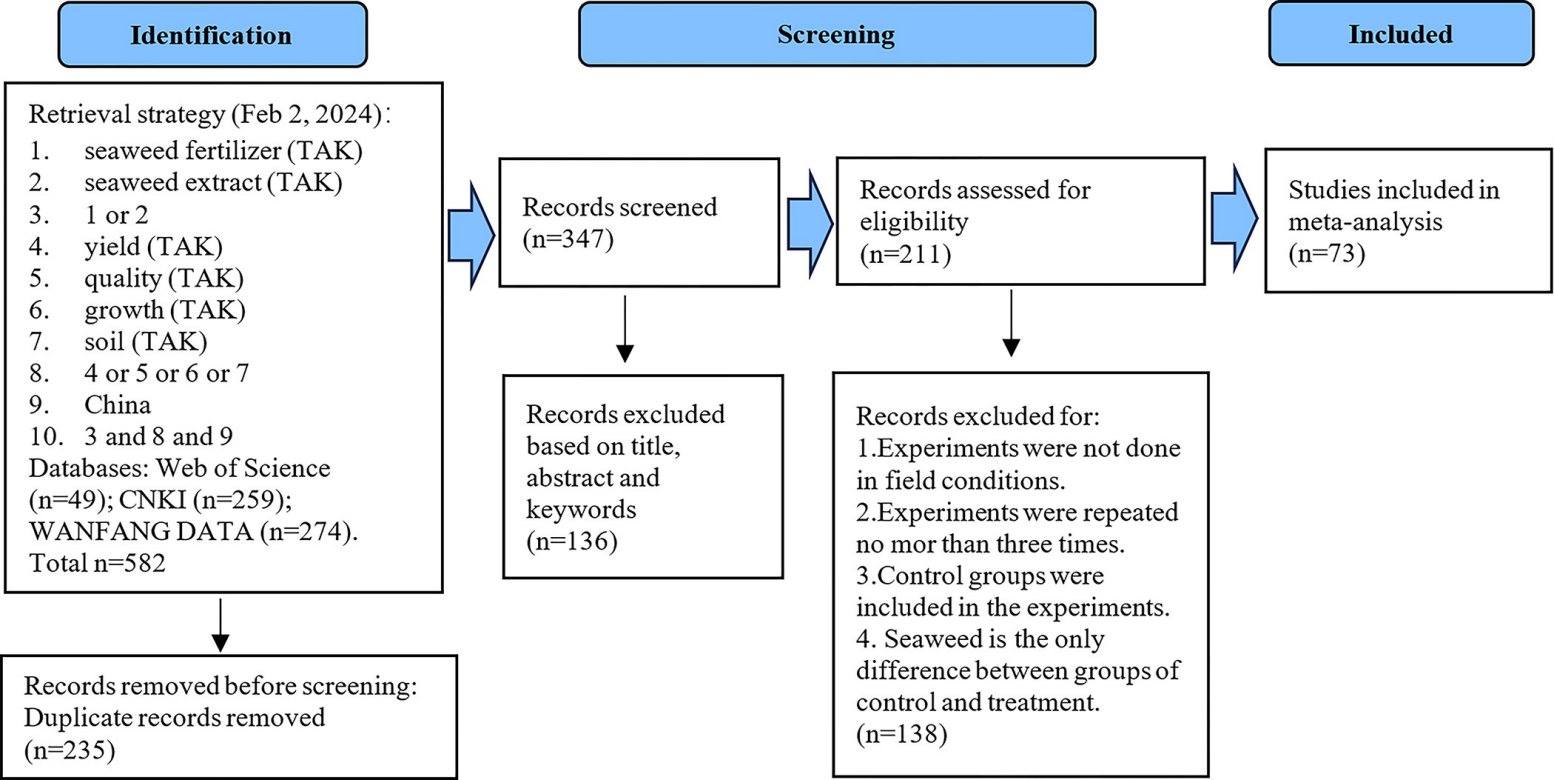
Identification of seaweed fertilizer studies via databases CNKI, China national knowledge infrastructure.

We followed the PRISMA 2020 statement for identification studies and conducted meta-analysis [25]. All relevant research studies were searched from Web of Science (https://webofscience.clarivate.cn), China National Knowledge Infrastructure (CNKI, https://www.cnki.net), and WANGFA DATA(http://www.wanfangdata.com) till Feb 2, 2024. Search terms are combinations of (‘seaweed fertilizer’ or ‘seaweed extract’) and (‘yield’ or ‘quality’ or ‘growth’ or ‘soil’) and China. The additional filter for studies is that, they must be from journals, specifically research papers rather than reviews. Subsequently, we obtained 582 studies in total, of which 49, 259, and 274 records are from the Web of Science, CNKI, and WANGFA DATA, respectively. First, duplicate records were removed using literature management software (Endnote X9, Thomson ResearchSoft, Stanford, CA, USA). Second, the records were screened based on the title, abstract, and keywords. Finally, studies were selected for further analysis with the following criteria. i) Experiments were conducted in field conditions, and locations were clear. ii) Experiments were repeated at least three times. iii) Control groups were included in the experiments, and seaweed was the only difference between the control and treatment groups. This means that if the seaweed fertilizer used in the treatment group was compounded with chemical or organic materials, the control group should be treated with the same type and quantity of chemical or organic materials. Finally, 73 studies were selected for further analysis (Fig 1 and S1 Table).

### Data collection

Microsoft Office Excel 2019 was used to create a database for the meta-analysis. Data collected from studies contain, i) study site information, including the experiment site’s locations, climate, soil pH, etc. ii) seaweed production and application information, including seaweed extract preparation, application methods, and crops. iii) Experiment information, including data of control and seaweed treatment, repeated times. The form of experimental data inputted into the database were means, Standard Deviation (SD), and repeat times in the control and seaweed treatments, respectively. If the experiment error was shown in Variance(S^2^) or Standard Error (SE), SD was calculated as $SD=SE\sqrt{n}$, where n represents the number of repeats. If the experiment was repeated three times or more, but the SD was not shown, the SD was calculated by multiplying the means by 0.1 [6,26]. We used GetData Graph Digitizer 2.25 (http://www.getdatagraphdigitizer.com/ index.php) to obtain data from the figures if they were not shown in the tables directly.

Crops were classified as cereals (maize, rice, etc.), root & tuber crops (potatoes and sweet potatoes, etc.), fruits (peach, strawberry, apple, etc.), vegetables (tomato, Chinese cabbage, etc.), oil crops (peanut, soybean, etc.), and other cash crops (cotton, flowers, etc.). The climate of the locations was classified as temperate continental climate, temperate monsoon climate, subtropical monsoon climate, plateau mountain climate, and tropical monsoon climate [27].

### Meta-analysis

#### Effect size and variance calculation

The effect size and variance of the seaweed fertilizer in each independent study were calculated using the MetaWin statistical software package Version 3.0.14 [28].

The effect size is represented in the natural log of the response ratio (*R*).


$$\ln\;R=\ln\frac{X_{t}}{X_{c}}$$ (1)

where *X*_t_ and *X*_c_ represent the means of the treatment groups and the mean of the control groups. Accordingly, a positive ln*R* indicates the positive effects of seaweed fertilizer on crop yield, quality, and growth, and a negative ln*R* indicates the negative effects. The variance of effect size(*V*_lnR_) and weight(*W*_i_) of each independent study was calculated according to Ma et al., 2021 [29] and Rosenberg, 2024 [28].


$$V_{\ln R}=\frac{S_{t}^{2}}{{N_{t}X}_{t}^{2}}+\frac{S_{c}^{2}}{N_{c}X_{c}^{2}}$$ (2)

$$W_{i}=1/V_{i}$$ (3)

Where *N*_t_ and *N*_c_ represent the sample sizes of the treatment groups and the control groups, *S*_t_ and *S*_c_ represent the standard deviations of the treatment and control groups.

#### Meta-analysis and heterogeneity test

The weighted mean effect size ln*R*_++_ and their variance were calculated in MetaWin software with fixed model first according to Rosenberg, 2024 [28].


$$\ln\;R_{++}=\sum_{i=1}^{k}W_{i}\;\ln\;R_{i}∕\sum_{i=1}^{k}W_{i}$$ (4)

$$V_{lnR++}=1/\Sigma w_{i}$$ (5)

The 95% confidence interval (Cl) of ln*R*_++_ was calculated as follows:

$${lnR}_{++}\pm 1.96\times S_{{lnR}_{++}}$$ (6)

Whereas the standard error $S_{{lnR}_{++}}$ was calculated as

$$S_{{lnR}_{++}}=\sqrt{1/\Sigma w_{i}}$$ (7)

If the 95% Cl did not cross the zero line, the seaweed fertilizer’s effect was regarded as significant (*P*<0.05) compared with the control [30]. If not, the effect was considered as insignificant.

*Q* Value (the statistic value of heterogeneity for explaining variance), degrees of freedom(df), and *I*^2^ (the variation in ln*R* attributable to heterogeneity) were calculated to show the heterogeneity of the group data.

$$Q=\Sigma W_{i}{\left(lnR-\bar{lnR}\right)}^{2}$$ (8)

df=N‐1 (9)

$$I^{2}=max\left[0,100\times \frac{Q_{B}-(n-1)}{Q_{B}}\right]$$ (10)

where the $\bar{\mathrm{lnR}}$ represents the mean of ln*R*, and N represents the number of studies. If the *Q*<df, *I*^2^<50, and *P*>0.05, it means the data heterogeneity is relatively low, and the independent variable had a relative fix effect on the dependent variable. Thus, the data is appropriate for further analysis with fix model. If not, the data heterogeneity is relatively high, and the independent variable may have a relative random effect on the dependent variable. Therefore, a random model is more appropriate in further meta-analysis. In the random model, the variation *V*′_*i*_ and weight $W_{i}^{\prime }$ were adjusted as

$${V\prime }_{i}=\frac{Q-(n-1)}{\Sigma w_{i}-\Sigma w_{i}^{2}/\Sigma w_{i}}$$ (11)

$$W_{i}^{\prime }=1/\left(V_{i}+V_{i}^{2}\right)$$ (12)

Then the *lnR*_++_ was recalculated with adjusted weight $W_{i}^{\prime }$. Moreover, in some situations, the data need to be divided into subgroups on the basis of their properties or run the sensitivity analysis to find and delete the exceptional data [31,32]. In this study, heterogeneity is relatively high in the fix effect size model, so the random model was chosen for meta-analysis (S2 Table).

#### Publication bias test

To test the publication bias of the data, Rosenthal’s Fail-safe Number and Egger regression were calculated based on the effect sizes of seaweed fertilizer application, regardless the crops types, using method or locations et al. If the Fail-safe Number is greater than 5N + 10 (N is the number of observations included in this study), there is no notable publication bias [6,33]. If the Egger regression *P* value is greater than 0.05, there is no notable publication bias [34]. If there was publication bias in the study, further analysis (such as Trim and fill analysis) was needed to address the bias and adjust the results of analysis [35].

#### Statistics

In the meta-analysis process, if the effect sizes of seaweed fertilizer application (regardless the crops types, using method or locations et al.) were not distributed normally, the sampling method option ‘bootstrapping (resampling nonparametric estimation method)’ should be selected [36]. To test the normality of the seaweed fertilizer effect size in this study, we calculated the seaweed fertilizer effect size distribution frequency and fitted them with gaussian function in SPSS 25.0 statistics software (IBM SPSS, Inc, Armonk, NY, USA). Moreover, a Q-Q plot and Kolmogorov-Smirnov test were employed to confirm the normality in SPSS. OriginPro 2022 SR1 9.9.0.225 (OriginLab Corporation, Massachusetts, USA) was used to make the figures.

## Results

### Heterogeneity test

In the meta-analysis, the variation of individual study effect sizes results in statistical heterogeneity. If the data heterogeneity is high, it needs to be addressed for further analysis[37]. In this study, the heterogeneity is relatively low in the data of seaweed fertilizer effects on yield increase. *Q* is not much higher than df, *P(*χ^2^) is not less than 0.05, and *I*^2^ is much lower than 50 (Table 1).

**Table 1:** Group data heterogeneity of different effects of seaweed fertilizer.

Seaweed fertilizer effects	*Q*	df	*P(*χ^2^)	*I* ^2^
**Yield increasing**	82.47	71	0.17	13.91
**Growth improvement**	158.52	106	0.01	33.13
**Quality improvement**	143.19	97	0.00	32.26

*Q*, the statistic value of heterogeneity for explaining variance; df, the degree of freedom; *P*(χ2), the significant value of heterogeneity for explaining variance; *I*^2^, the variation in ln*R* attributable to heterogeneity, the same as following Table 2.

However, for the effects of seaweed fertilizer on crop quality and growth improvement, the heterogeneity is relatively high (Table 1). To determine the source of high heterogeneity, we run a subgroup analysis based on data properties. In further subgroup analysis, the heterogeneity of all quality factors was relatively low and appropriate for further analysis (Tables 2 and 3).

**Table 2:** Group data heterogeneity of different quality improvement effects of seaweed fertilizer.

Quality improvement effects	*Q*	df	*P(*χ^2^)	*I* ^2^
**Fruit hardness**	5.87	6	0.44	0.00
**Protein**	2.93	5	0.71	0.00
**Reducing sugar**	1.88	3	0.59	0.00
**Soluble solid**	15.57	16	0.48	0.00
**Soluble sugar**	7.48	13	0.88	0.00
**Starch**	1.87	3	0.60	0.00
**Sugar-acid ratio**	8.93	7	0.26	21.64
**Titratable acid**	7.27	10	0.70	0.00
**Total sugar**	18.34	11	0.07	40.03
**Vitamin C**	17.65	14	0.22	20.68

**Table 3:** Group data heterogeneity of different growth improvement effects of seaweed fertilizer.

Growth improvement effects	*Q*	df	*P(*χ^2^)	*I* ^2^
**Height**	37.06	31	0.21	16.35
**Leaves number**	2.16	3	0.54	0.00
**Leaves area**	9.85	6	0.13	39.07
**Leaves width**	6.60	7	0.47	0.00
**Leaves length**	5.72	4	0.22	30.12
**DBH**	8.11	12	0.78	0.00
**Root weight**	2.52	2	0.28	20.74
**Photosynthetic rate**	15.19	10	0.13	34.17
**Chlorophyll**	32.58	21	0.05	35.56

### Distribution of effect sizes of seaweed fertilizer application

**Fig 2:**
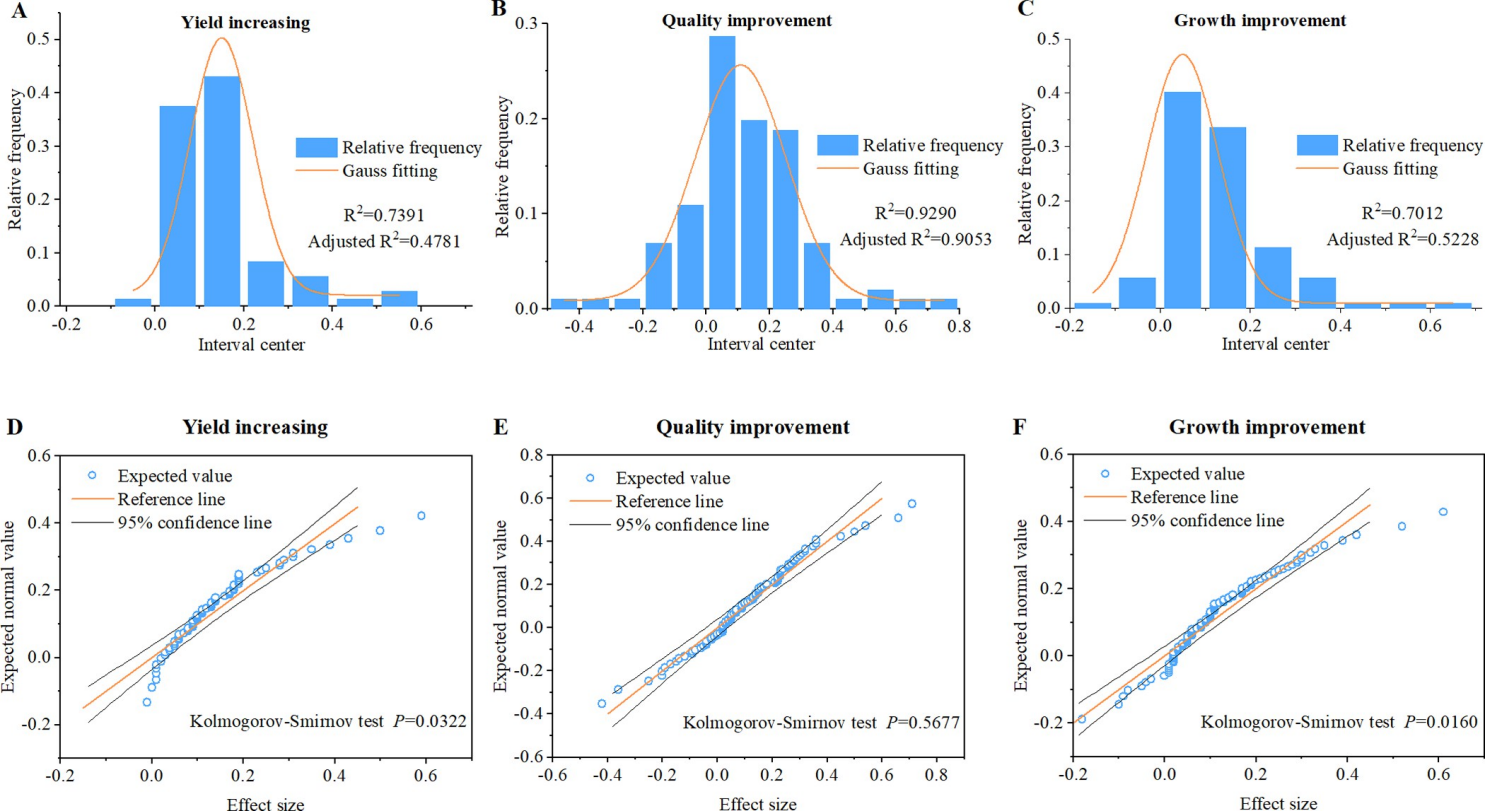
Frenquency distribution fitting, plotting and test of seaweed fertilizer effect on crops’ yield increasing, quality and growth improvement. (A), (B) and (C), Frenquency distribution with gauss fitting of seaweed fertilizer effect on crops’ yield increasing, quality, and growth improvement, respectively; (D), (E) and (F), Q-Q plotting, and Kolmogorov-Smirnov test of seaweed fertilizer effect on crops’ yield increasing, quality, and growth improvement, respectively.

Before the conducton of meta-analysis, whether the data distribution is normal or not should be clear. Gauss function fitting of seaweed fertilizer effect sizes showed that, quality improvement effect sizes of seaweed fertilizer distributed relatively normally with an adjusted R squared of 0.91, wheras a relatively abnormal distribution of yield increasing and growth improvement effect size was observed with an adjusted R squared of 0.48 and 0.52, respectively (Fig 2A–2C).

Moreover, Q-Q plotting and the Kolmogorov-Smirnov test on seaweed fertilizer effect sizes indicated similar results. It was show that the seaweed fertilizer effects on quality improvement distributed normally in the Q-Q plot, and *P* values were greater than 0.05 (Fig 2E). In contrast, seaweed fertilizer effects of yield increase and growth improvement did not distribute normally in the Q-Q plot, and *P* values were less than 0.05 in the Kolmogorov-Smirnov test (Fig 2D and 2F). Thus, we selected the bootstrapping method in meta-analysis for yield increase and growth improvement effect of seaweed fertilizer.

### Effects of seaweed fertilizer application on crops yield and quality in field conditions in China

**Fig 3:**
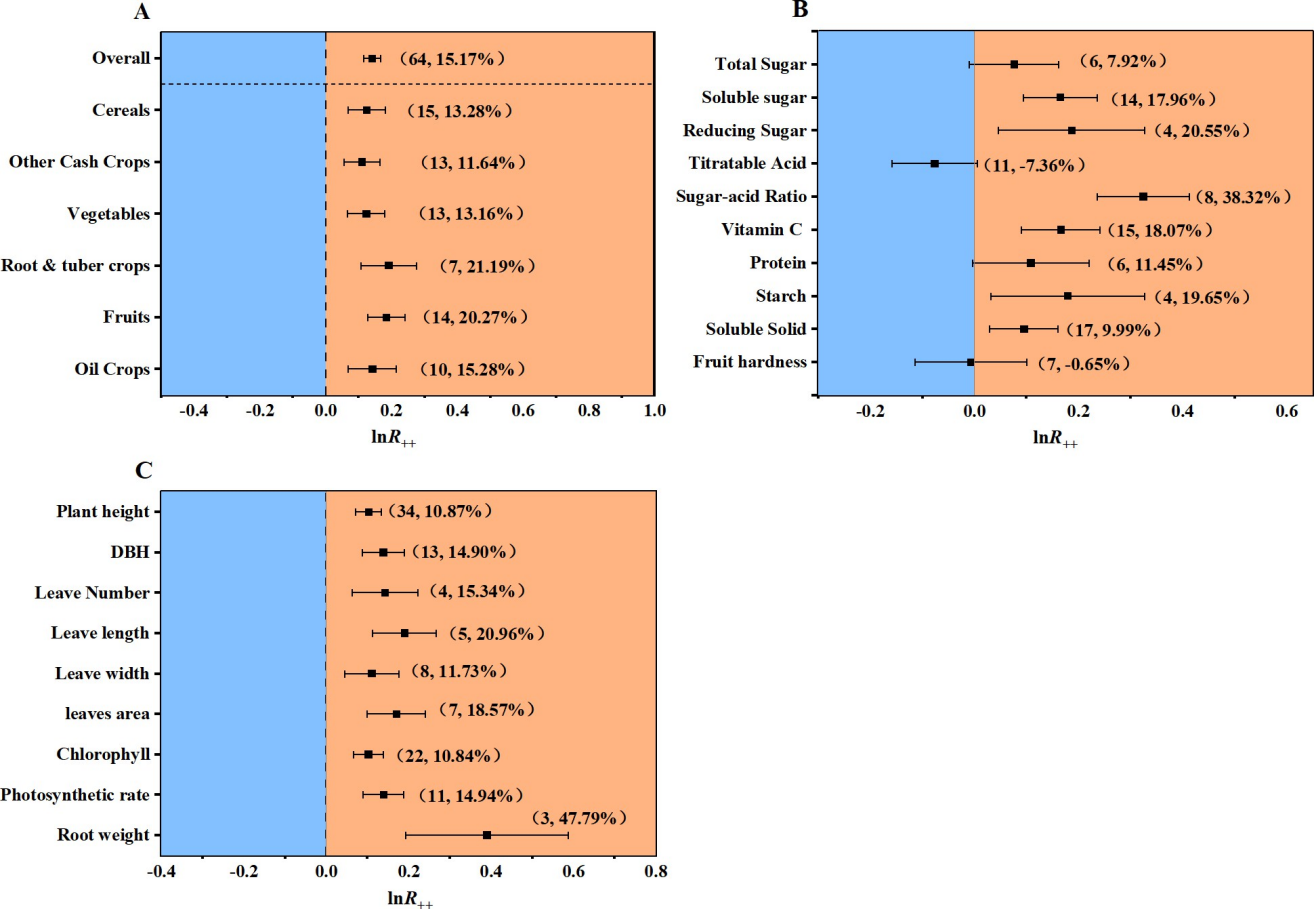
Effects of seaweed fertilizer on crops’ yield, quality, and growth. (A) Effects of seaweed fertilizer on crops’ yield; (B) Effects of seaweed fertilizer on crops’ quality. (C) Effects of seaweed fertilizer on crops’ growth. All the first numbers in parentheses of (A), (B), and (C) represent the studies numbers, and the second ones indicated the change rates of each factor under seaweed fertilizer applications to controls, the same as following Fig 4.

The results of the meta-analysis indicated that the mean of all crops’ yield increased by 15.17% significantly with seaweed fertilizer than without seaweed fertilizer (Fig 3A). Compared with the control, the yield of root & tuber crops and fruits was notably higher when seaweed fertilizer was applied, with an increase of 21.19% and 20.27%, respectively. Following were oil crops, cereals, vegetables, and other cash crops, with significant increase of 15.28%, 13.28%, 13.16%, and 11.64%, respectively (Fig 3A). Much more interesting, seaweed fertilizer application improved the crop yield in all catalogs significantly in statistics (95% confidence intervals didn’t cross the zero-line, Fig 3A).

The quality of the crop was also improved with seaweed fertilizer application. Compared with the control, total sugar, soluble sugar, and reducing sugar increased by 7.92%, 17.96%, and 20.55%, respectively (Fig 3B). Hence, the crops’ sugar content was increased. Conversely, the titratable acid significantly decreased by 7.36%. Consistent with these changes, the sugar-acid ratio, which is important for the flavor of fruits and vegetables, was significantly increased by 38.32% (Fig 3B). Moreover, seaweed fertilizer application significantly improved the crops’ starch, vitamin C, protein, and soluble solid by 19.65%, 18.07%, 11.45%, and 9.99%, respectively (Fig 3B). These effects also benefit crop qualities. In addition, increasing soluble solids contribute to the high quality of some fruits or vegetables.

Furthermore, the growth characteristics of crops under seaweed fertilization were also improved. Compared with the control, the growth of root, stem, leaf, and plant height improved due to seaweed fertilizer application. For instance, plant height and diameter at breast height (DBH) increased by 10.87% and 14.90% (Fig 3C). Notably, root weight increased by 47.79%. Compared with the control, seaweed fertilizer application significantly improved leaf growth characteristics, including the number, length, width, and area of leaves, with improvements of 15.34%, 20.96, 11.73%, and 18.57%, respectively (Fig 3C). Moreover, Chlorophyll content in leaves increased by 10.84%. Meanwhile, the photosynthetic rate increased by 14.19% (Fig 3C).

### Impact factors of seaweed fertilizer on crops yield in field conditions in China

**Fig 4:**
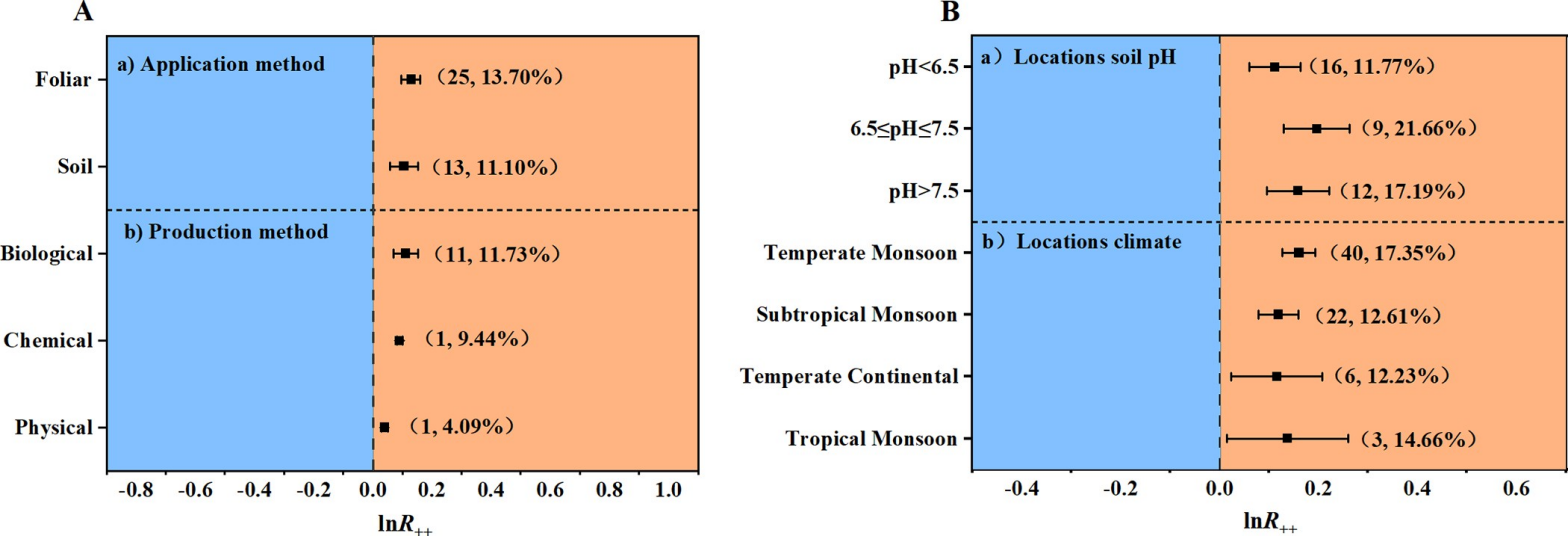
The effect of seaweed fertilizer on crops’ yield increasing varied on its preparation, application, and use locations. (A) The effect size of seaweed fertilizer on crops’ yield increasing with different methods of preparation and application. (B) Effect size of seaweed fertilizer on crops’ yield increasing in different locations soil pH and in different climate zones.

To further clarify the effects of seaweed fertilizer on crop yield, a meta-analysis was conducted on factors that could impact the effect of seaweed fertilizer on crops, including the methods and situations in seaweed fertilizers production and application processes. First, for seaweed extract extraction, analysis results showed that seaweed fertilizer made based on biological extraction significantly increased crops’ yield by 11.73%, whereas that based on physical or chemical extraction also increased crops’ yield, but much lower in statistics, which is 4.09% and 9.44% (Fig 4A). Second, for the seaweed fertilizer application process, foliar and root application significantly increased crop yield by 13.70% and 11.10%, respectively (Fig 4A). Third, for locations of seaweed fertilizer application, the analysis results indicated that climate and soil pH both impacted the effects of seaweed fertilizer. In the regions with temperate monsoon and tropical monsoon climates, crop yield increased significantly by 17.35% and 14.66% under seaweed fertilizer application, respectively. In the regions of temperate continental and subtropical monsoon climate, the yield also significantly increased by 12.23% and 12.61%, respectively (Fig 4B). Moreover, the analysis results show that compared with the control, seaweed fertilizer application increased crop yield significantly in the field with a wide range of soil pH, with the highest increment in neutral soil (21.66%, pH 6.5–7.5), followed by in relatively alkaline soil (17.19%, pH>7.5) and relatively acidic soil (11.77%, pH<6.5, Fig 4B).

### Publication bias test

Publication bias is common in meta-analysis, because positive and significant results are easier to publish. When negative or nonsignificant research results are unpublished, publication bias occurs. In this study, publication bias was tested by the Fail-safe Number and egger regression. If the number of the studies is greater than 5N + 10 (N is the number of observations included in this study), there is no notable publication bias. We ran Rosenthal’s Fail-safe Number test in the seaweed fertilizer effect of yield increase, quality, and growth improvement, which were 2300.88, 1283.97, and 5107.27, respectively (Table 4). All of them are greater than 5N + 10 (N is 72, 98, and 107, respectively in this study), which indicates that there is no notable publication bias in this study.

**Table 4:** Fail-safe number test of seaweed fertilizer effect on crops’ yield increasing, quantity and growth improvement.

Seaweed fertilizer effect	Yield increasing	Quality improvement	Growth improvement
**Rosenthal’s Fail-safe Number**	2300.88	1283.97	5107.27
**Study number (N)**	72	98	107
**‘5N+10’**	370	500	545

**Fig 5:**
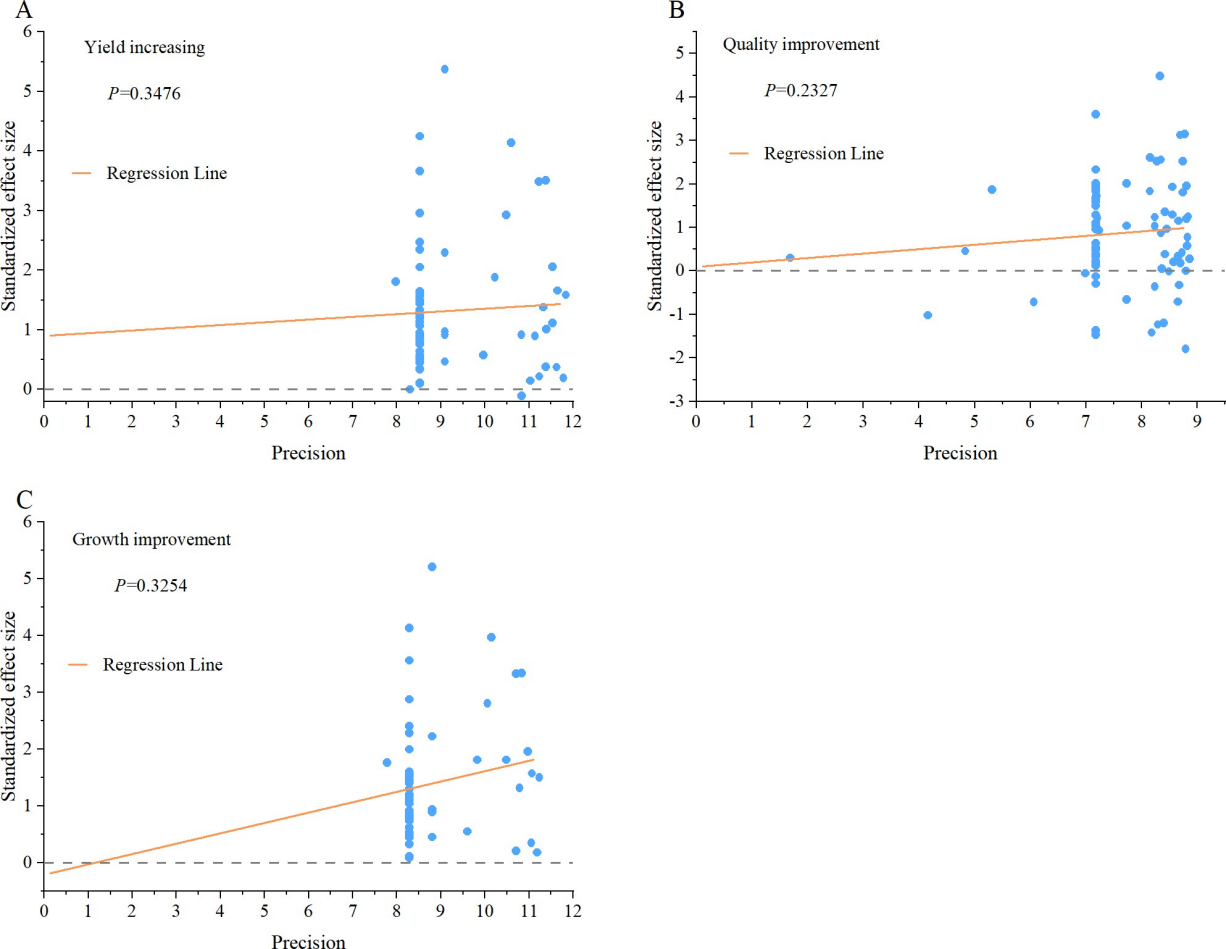
Egger regression of seaweed fertilizer effects on crop yield, quality, and growth Nattier blue solid circles represent the crossover point standized effect size and its precision.

Moreover, we conducted an Egger regression test. In the test, if the *P* value is greater than 0.05, there is no notable publication bias. Seaweed fertilizer effect size (ln*R*) of yield increase, quality, and growth improvement were tested, and the *P* values were 0.3476, 0.2327, and 0.3254, respectively (Fig 5). Results also indicated that no notable publication bias was observed in this study. These results indicate that our meta-analysis results in this study can be considered robust and reliable.

## Discussion

In recent decades, extensive nutrient management led to yield stagnation, declining quality, declining soil condition and environmental pollution in China [3–5]. These are big challenges for sustainable agriculture. Therefore, many projects, including the chemical fertilizer ‘Zero Increase Action Plan’ were carried out in China [6]. In this study, crop yield increased by 15.17% with seaweed fertilizer application. Furthermore, crop quality was also improved after seaweed fertilizer application. Quality indexes such as the contents of protein, vitamin C, and sugar increased, whereas the titratable acid content decreased (Fig 3A and 3B). These content changes may lead to a better taste of fruits or vegetables. As indicated above, seaweed fertilizer could be a feasible alternative to chemical fertilizer.

Compared with continental plants, seaweed contains not only common continental mineral nutrients or small molecular organic nutrients but also unique marine mineral nutrients and bioactive compounds. The concentrations of mineral, trace, and ultra-trace elements in seaweeds are higher than those in terrestrial plants [38]. Several plant hormones (such as auxin, cytokinin, betaine, etc.) were found in seaweed [11,39]. Therefore, seaweed fertilizer could provide plants with both nutrients and stimulators for their growth. In this study, we found that plants’ height, DBH, root weight, leaf area, etc. all increased after fertilization with seaweed fertilizer (Fig 3C). Moreover, in addition to several mineral elements, proteins, anomic acids, and vitamins, seaweed also contains a certain amount of polysaccharide, whose main form is alginic acid [40]. Alginic acid provides plants with energy and strengthens plant’s cell walls [8,41]. Alginate oligosaccharides, which are degradations from polysaccharides, are nutrients and stimulators for plants. They are small molecular weights, water-soluble, and easily absorbed by plants [42,43]. Furthermore, soil enzymes, including soil sucrase, urease, soil glycosidase activities, and soil microbe community diversity could be improved by seaweed fertilizer application, and these changes were considered responsible for the stress tolerances of plants [15,20]. These results were consistent with the analysis results in this study, which showed that seaweed fertilizer had positive effects on crop yield regardless of soil pH and the climate types of site locations.

Compared with the clearly defined nutrient composition of chemical fertilizers, seaweed fertilizers have a complex nutrient profile, making it challenging to tailor customized formulations. Additionally, higher application technology is required for seaweed fertilizers to achieve optimal effectiveness. Specifically, large number of seaweed species, different methods of extraction, production, and application diverse the effects of seaweed fertilizer in field conditions. According to their color, Seaweeds were divided into three categories (red, brown, and green). More specifically, the number of known seaweed species is up to 11,000 [44]. The concentrations of bioactive matter and metabolites are diverse in different seaweed species [45]. Furthermore, these seaweeds were extracted through distinctive methods to produce seaweed fertilizer, which was then applied specifically way at different locations. Therefore, the effects of the seaweed fertilizer varied. In this study, analysis data showed that the yield-increasing effect of seaweed fertilizer was relatively high when the seaweed extraction was performed using biological methods rather than those through chemical or physical methods (Fig 4A). Compared with other conditions, seaweed fertilizer performed better in neutral and alkaline soils, within temperate monsoon and tropic monsoon climates. However, in all locations within this study, seaweed fertilizer increased the yield significantly (Fig 4B). It might be related to the function of seaweed fertilizer in improving plant stress tolerance and soil properties [46–48].

For meta-analysis, it is difficult to obtain statistical results without any bias and limitations. In this study, we sought data heterogeneity and addressed it (Tables 1–3), found an unnormal distribution of data, chose the appropriate sampling method (Fig 2), and tested the potential of publication bias (Table 4 and Fig 5). Regardless of the inherent limitations of the statistical process, our results are robust and reliable, and could be a useful reference for seaweed fertilizer research and application.

## Conclusions

We conducted a meta-analysis on the effect of seaweed fertilizer in field conditions in China. Results showed that seaweed fertilizer application increased the crops’ yield. On average, there was a 15.17% increase in crop yield. Additionally, regarding crop quality, the application of seaweed fertilizer resulted in an increase in sugar content and a decrease in titratable acidity, leading to a significant rise in the sugar-acid ratio by 38.32%. Furthermore, the crops vitamin C, protein, starch, soluble solids, etc., showed significant improvements. These findings suggest that seaweed fertilizer could be a suitable option for sustainable agriculture in China. Specifically, the effectiveness of seaweed fertilizer in increasing yields varied depending on its preparation methods, application techniques, and regions. Seaweed fertilizer produced using biological methods and applied in neutral soil exhibited a relatively stronger effect on increasing yields.

## Supporting information

S1 Table Identified studies for meta-analysis in this study. (DOCX)

S2 Table Group data heterogeneity of different effects of seaweed fertilizer with fix model in meta-analysis. (DOCX)

## References

[1] FAOSTAT. Population and Land Use 2023 [2023-04-08]. Available from: http://www.fao.org/faostat/en/.

[2] Song X, Yang LE, Xia F, Zhao G, Xiang J, Scheffran J. An inverted U-shaped curve relating farmland vulnerability to biological disasters: Implications for sustainable intensification in China. The Science of the total environment. 2020; 732:138829. doi: 10.1016/j.scitotenv.2020.138829 32438151

[3] Wang J, Qin L, Cheng J, Shang C, Li B, Dang Y, et al. Suitable chemical fertilizer reduction mitigates the water footprint of maize production: evidence from Northeast China. Environmental science and pollution research international. 2022;29(15):22589–601. doi: 10.1007/s11356-021-17336-2 34792771

[4] Xu W, Liu W, Tang S, Yang Q, Meng L, Wu Y, et al. Long-term partial substitution of chemical nitrogen fertilizer with organic fertilizers increased SOC stability by mediating soil C mineralization and enzyme activities in a rubber plantation of Hainan Island, China. Applied Soil Ecology. 2023; 182:104691. 10.1016/j.apsoil.2022.104691.

[5] Fan Q, Xu C, Zhang L, Xie J, Zhou G, Liu J, et al. Application of milk vetch (Astragalus sinicus L.) with reduced chemical fertilizer improves rice yield and nitrogen, phosphorus, and potassium use efficiency in southern China. European Journal of Agronomy. 2023; 144:126762. 10.1016/j.eja.2023.126762.

[6] Du Y, Cui B, zhang Q, Wang Z, Sun J, Niu W. Effects of manure fertilizer on crop yield and soil properties in China: A meta-analysis. CATENA. 2020; 193:104617. 10.1016/j.catena.2020.104617.

[7] Katakula AAN, Gawanab W, Itanna F, Mupambwa HA. The potential fertilizer value of Namibian beach-cast seaweed (Laminaria pallida and Gracilariopsis funicularis) biochar as a nutrient source in organic agriculture. Scientific African. 2020;10: e00592. 10.1016/j.sciaf.2020.e00592.

[8] Meng C, Gu X, Liang H, Wu M, Wu Q, Yang L, et al. Optimized preparation and high-efficient application of seaweed fertilizer on peanut. Journal of Agriculture and Food Research. 2022;7:100275. 10.1016/j.jafr.2022.100275.

[9] Larrea-Marín MT, Pomares-Alfonso MS, Gómez-Juaristi M, Sánchez-Muniz FJ, de la Rocha SR. Validation of an ICP-OES method for macro and trace element determination in Laminaria and Porphyra seaweeds from four different countries. Journal of Food Composition and Analysis. 2010;23(8):814–20. 10.1016/j.jfca.2010.03.015.

[10] Astorga-España MS, Rodríguez Galdón B, Rodríguez Rodríguez EM, Díaz Romero C. Mineral and trace element concentrations in seaweeds from the sub-Antarctic ecoregion of Magallanes (Chile). Journal of Food Composition and Analysis. 2015; 39:69–76. 10.1016/j.jfca.2014.11.010.

[11] Valencia RT, Acosta LS, Hernández MF, Rangel PP, Agronomy CVVJ. Effect of Seaweed Aqueous Extracts and Compost on Vegetative Growth, Yield, and Nutraceutical Quality of Cucumber (Cucumis sativus L.) Fruit. 2018;8(11):264. 10.3390/agronomy8110264.

[12] Battacharyya D, Babgohari MZ, Rathor P, Prithiviraj B. Seaweed extracts as biostimulants in horticulture. Scientia Horticulturae. 2015; 196:39–48. 10.1016/j.scienta.2015.09.012.

[13] Papenfus HB, Kulkarni MG, Stirk WA, Finnie JF, Van Staden JJeH. Effect of a commercial seaweed extract (Kelpak®) and polyamines on nutrient-deprived (N, P and K) okra seedlings. 2013; 151:142–6. 10.1016/j.scienta.2012.12.022.

[14] Cabo S, Morais MC, Aires A, Carvalho R, Pascual-Seva N, Silva AP, et al. Kaolin and seaweed-based extracts can be used as middle and long-term strategy to mitigate negative effects of climate change in physiological performance of hazelnut tree. Journal of Agronomy and Crop Science. 2020;206(1):28–42. 10.1111/jac.12369.

[15] Chen D, Li Z, Yang J, Zhou W, Wu Q, Shen H, et al. Seaweed extract enhances drought resistance in sugarcane via modulating root configuration and soil physicochemical properties. Industrial Crops and Products. 2023; 194:116321. 10.1016/j.indcrop.2023.116321.

[16] du Jardin P. Plant biostimulants: Definition, concept, main categories and regulation. Scientia Horticulturae. 2015; 196:3–14. 10.1016/j.scienta.2015.09.021.

[17] Civelek Yoruklu H, Ozkaya B, Demir A. Optimization of liquid fertilizer production from waste seaweed: A design of experiment based statistical approach. Chemosphere. 2022;286(Pt 3):131885. doi: 10.1016/j.chemosphere.2021.131885 34411930

[18] Wang M, Chen L, Li Y, Chen L, Liu Z, Wang X, et al. Responses of soil microbial communities to a short-term application of seaweed fertilizer revealed by deep amplicon sequencing. Applied Soil Ecology. 2018; 125:288–96. 10.1016/j.apsoil.2018.02.013.

[19] Zhou G, Qiu X, Zhang J, Tao C. Effects of seaweed fertilizer on enzyme activities, metabolic characteristics, and bacterial communities during maize straw composting. Bioresource technology. 2019; 286:121375. doi: 10.1016/j.biortech.2019.121375 31030066

[20] Wang Y, Fu F, Li J, Wang G, Wu M, Zhan J, et al. Effects of seaweed fertilizer on the growth of Malus hupehensis Rehd. seedlings, soil enzyme activities and fungal communities under replant condition. European Journal of Soil Biology. 2016; 75:1–7. 10.1016/j.ejsobi.2016.04.003.

[21] Bank World. Global Seaweed: New and Emerging Markets Report, 2023. Washington, DC: World Bank, 2023. http://hdl.handle.net/10986/40187.

[22] Chatterjee A, Singh S, Agrawal C, Yadav S, Rai R, Rai LC. Chapter 10—Role of Algae as a Biofertilizer. In: Rastogi RP, Madamwar D, Pandey A, editors. Algal Green Chemistry. Amsterdam: Elsevier; 2017. p. 189–200.

[23] Belghit I, Rasinger JD, Heesch S, Biancarosa I, Liland N, Torstensen B, et al. In-depth metabolic profiling of marine macroalgae confirms strong biochemical differences between brown, red and green algae. Algal Research. 2017;26:240–9. 10.1016/j.algal.2017.08.001.

[24] Williams TI, Edgington S, Owen A, Gange AC. Evaluating the use of seaweed extracts against root knot nematodes: A meta-analytic approach. Applied Soil Ecology. 2021; 168:104170. doi: 10.1016/j.apsoil.2021.104170 34866802 PMC8501307

[25] Page MJ, McKenzie JE, Bossuyt PM, Boutron I, Hoffmann TC, Mulrow CD, et al. The PRISMA 2020 statement: An updated guideline for reporting systematic reviews. PLOS Medicine. 2021;18(3):e1003583. doi: 10.1371/journal.pmed.1003583 33780438 PMC8007028

[26] Rose MT, Patti AF, Little KR, Brown AL, Jackson WR, Cavagnaro TR. Chapter two—A meta-analysis and review of plant-growth response to humic substances: practical implications for agriculture. In: Sparks DL, editor. Advances in Agronomy. 124: Academic Press; 2014. p. 37–89. 10.1016/B978-0-12-800138-7.00002-4

[27] Li SK. Agroclimatic regionalization of China. Journal of Natural Resources. 1987; 1:71–83. 10.11849/zrzyxb.1987.01.008.

[28] Rosenberg MS. MetaWin 3: open-source software for meta-analysis. Frontiers in Bioinformatics. 2024; 4:1305969. doi: 10.3389/fbinf.2024.1305969 38390304 PMC10883383

[29] Ma D, Yin L, Ju W, Li X, Liu X, Deng X, et al. Meta-analysis of green manure effects on soil properties and crop yield in northern China. Field Crops Research. 2021; 266:108146. 10.1016/j.fcr.2021.108146.

[30] Hedges LV, Curtis GPSJE. The meta-analysis of response ratios in experimental ecology. Ecology. 1999;80(4):1150–6. 10.2307/177062.

[31] Higgins JP, Thompson SG. Quantifying heterogeneity in a meta-analysis. Statistics in Medicine. 2002;21(11):1539–58. doi: 10.1002/sim.1186 12111919

[32] Huedo-Medina TB, Sánchez-Meca J, Marín-Martínez F, Botella J. Assessing heterogeneity in meta-analysis: Q statistic or I2 index? Psychological methods. 2006;11(2):193–206. doi: 10.1037/1082-989X.11.2.193 16784338

[33] Rosenberg MS. The file-drawer problem revisited: a general weighted method for calculating fail-safe numbers in meta-analysis. Evolution; international journal of organic evolution. 2005;59(2):464–8. 10.1111/j.0014-3820.2005.tb01004.x. 15807430

[34] Egger M, Davey Smith G, Schneider M, Minder C. Bias in meta-analysis detected by a simple, graphical test. BMJ (Clinical research ed). 1997;315(7109):629–34. doi: 10.1136/bmj.315.7109.629 9310563 PMC2127453

[35] Duval S, Tweedie R. Trim and fill: A simple funnel-plot-based method of testing and adjusting for publication bias in meta-analysis. Biometrics. 2000;56(2):455–63. doi: 10.1111/j.0006-341x.2000.00455.x 10877304

[36] Yan F, Zhao H, Wu L, Huang Z, Niu Y, Qi B, et al. Basic Cognition of Melatonin Regulation of Plant Growth under Salt Stress: A Meta-Analysis. Antioxidants (Basel, Switzerland). 2022;11(8). doi: 10.3390/antiox11081610 36009327 PMC9405259

[37] Ruppar T. Meta-analysis: How to quantify and explain heterogeneity? European journal of cardiovascular nursing. 2020;19(7):646–52. doi: 10.1177/1474515120944014 32757621

[38] Kiuomars Rohani-Ghadikolaei, Eessa Abdulalian, Ng W-K. Evaluation of the proximate, fatty acid and mineral composition of representative green, brown and red seaweeds from the Persian Gulf of Iran as potential food and feed resources. Journal of Food Science and Technology. 2011;49(6):774–80. doi: 10.1007/s13197-010-0220-0 24293698 PMC3550831

[39] Craigie JS. Seaweed extract stimuli in plant science and agriculture. Journal of Applied Phycology. 2011. 10.1007/s10811-010-9560-4.

[40] Supraja KV, Behera B, Balasubramanian P. Efficacy of microalgal extracts as biostimulants through seed treatment and foliar spray for tomato cultivation. Industrial Crops and Products. 2020;151. 10.1016/j.indcrop.2020.112453.

[41] Suman S, Spehia RS, Sharma V. Productivity of capsicum as influenced by fertigation with chemical fertilizers and humic acid. Journal of Plant Nutrition. 2016;39(3):410–6. 10.1080/01904167.2015.1069338.

[42] Zhang C, Wang W, Zhao X, Wang H, Yin H. Preparation of alginate oligosaccharides and their biological activities in plants: A review. Carbohydrate Research. 2020; 494:108056. doi: 10.1016/j.carres.2020.108056 32559511

[43] Guo Y, Huang G, Wei Z, Feng T, Zhang K, Zhang M, et al. Exogenous application of coronatine and alginate oligosaccharide to maize seedlings enhanced drought tolerance at seedling and reproductive stages. Agricultural Water Management. 2023; 279:108185. 10.1016/j.agwat.2023.108185.

[44] Park E, Yu H, Lim J-H, Hee Choi J, Park K-J, Lee J. Seaweed metabolomics: A review on its nutrients, bioactive compounds and changes in climate change. Food Research International. 2023; 163:112221. doi: 10.1016/j.foodres.2022.112221 36596150

[45] Hamid SS, MasatakaIchihara, KensukeSakurai, KatsutoshiAshino, YujinKadowaki, RieSoga, TomoyoshiTomita, Masaru. Metabolome profiling of various seaweed species discriminates between brown, red, and green algae. Planta. 2019;249(6). 10.1007/s00425-019-03134-1. 30891648

[46] Shang X-c, Zhang M, Zhang Y, Li Y, Hou X, Yang L. Combinations of waste seaweed liquid fertilizer and biochar on tomato (Solanum lycopersicum L.) seedling growth in an acid-affected soil of Jiaodong Peninsula, China. Ecotoxicology and Environmental Safety. 2023;260:115075. doi: 10.1016/j.ecoenv.2023.115075 37267778

[47] Wang Y, Xiang L, Wang S, Wang X, Chen X, Mao Z. Effects of seaweed fertilizer on the Malus hupehensis Rehd. seedlings growth and soil microbial numbers under continue cropping. Acta Ecologica Sinica. 2017;37(3):180–6. 10.1016/j.chnaes.2017.01.004.

[48] Ahmed M, Ullah H, Piromsri K, Tisarum R, Cha-um S, Datta A. Effects of an Ascophyllum nodosum seaweed extract application dose and method on growth, fruit yield, quality, and water productivity of tomato under water-deficit stress. South African Journal of Botany. 2022; 151:95–107. 10.1016/j.sajb.2022.09.045.

