# Supplementary material for: Effects of seaweed fertilizer application on crops’ yield and quality in field conditions in China-A meta-analysis
Source: PLoS One. 2024 Jul 18;19(7):e0307517. doi: 10.1371/journal.pone.0307517 (PMC11257332; doi:10.1371/journal.pone.0307517)
Supplement: S1 Table — (DOCX) [file pone.0307517.s001.docx]

S1 Table Identified studies for meta-analysis in this study

| **No.** | **References(in Chinnese with English anstract)** |
| --- | --- |
| 1 | Wang, J.; Ding, Y.; Ye, X. L.; Fan, S. Y. Effects of different fertilizer treatments on yield and quality of broccoli. Acta Agriculturae Universitatis Jiangxiensis, 2013,35(3):517-520. doi: 10.3969/j.issn.1000-2286.2013.03.015 |
| 2 | Wang, Y. X.; Li, F. D.; Li, Y. J.; Zhang, F. X.; Sun, Q. T.; Zhang, X.; Tian, C. P. Effects of different kinds of foliar fertilizers on fruit quality of late ripening nectarine cultivar Fumei. Shandong Agricultural Sciences, 2018,50(9):48-50. doi: 10.14083/j.issn.1001-4942.2018.09.011 |
| 3 | Tang, H. J.; Xu, Y. H.; Yao, X. M.; Ma, Z. G.; Shen, Q. B.; Liu, J. Q.; Zhang, Y. L. Effects of different concentrations of seaweed fertilizer on yield and quality of purple sweet potato. Bulletin of Agricultural Science and Technology, 2014(11):49-51. doi: 10.3969/j.issn.1000-6400.2014.11.017 |
| 4 | Yan, F. G.; Li, S. D.; Tang, J. Effect of seaweed active substances extracted by different methods on soybean growth. Modern Agricultural Science and Technology, 2014(14):11-12. |
| 5 | Mou, X. L.; Zhu, G. L.; Dong, H.; Zhang, X. F.; Yu, S. H.; Shi, G. F.; Tan, D. S. Effects of different types of nitrogen fertilizer on summer maize yield, soil nutrients and fertilizer use efficiency. Shandong Agricultural Sciences, 2020,52(11):41-45. doi: 10.14083/j.issn.1001-4942.2020.11.008 |
| 6 | Ma, D.; Liu, H. Y.; Shan, J. W.; Tong, X. X.; Li, J. Q. Effects of mixed extracts of Laminaria japonica aresch and Enteromorpha prolifera on the growth and quality of strawberry. Soils and Fertilizers Sciences in China, 2017(5):129-134. doi:10.11838/sfsc.20170522 |
| 7 | Duan, Y. H.; Deng, C. Z.; Zuo, L. J.; Yin, J. F.; Bao, J. H.; Zhang, Y. R.; Yang, J. C.; Zhang, Z. Effects of different application amounts of seaweed fertilizer on yield and benefit of potato. Yunnan Agricultural Science and Technology, 2021(1):6-7. doi: 10.3969/j.issn.1000-0488.2021.01.003 |
| 8 | Zhang, Y.; Song, X. C.; Wei, T. Y.; Wang, H. L. Effects of seaweed fertilizer on the growth and development of peanut and soil physicochemical properties. South China Agriculture, 2021,15(20):4-6. doi: 10.19415/j.cnki.1673-890x.2021.20.002 |
| 9 | Jiang, X. L.; Xu, W. H. Yu, Z. F.; Chen, M.; Lin, W. B. Effects of seaweed fertilizer on yield, quality and resistance of cucumber. Vegetables, 2002(8):29-30. doi: 10.3969/j.issn.1001-8336.2002.08.021 |
| 10 | Li, J. C. Effects of seaweed fertilizer on fruit set and yield of apple. The Journal of Hebei Forestry Science and Technology, 2014(1):30-31,32. doi: 10.3969/j.issn.1002-3356.2014.01.014 |
| 11 | Ding, N. The research of seaweed fertilizer affecting on the quality and growth of pomegranate. Chinese Horticulture Abstract, 2018,34(5):15-16,40. doi: 10.3969/j.issn.1672-0873.2018.05.006 |
| 12 | Liu, J.; Zhao, L. L.; Feng, G.; Geng, Z. G.; Qin, Y. M. Analysis of grey correlation degree of seaweed fertilizer on growth characteristics of buckwheat. Seed Science & Technology, 2018,36(11):113-115. doi: 10.3969/j.issn.1005-2690.2018.11.084 |
| 13 | Huang, Q. M.; Xiao, Z. W.; Guan, J. J.; Zhang, P.; Zhang, H.; Wang, Y. L.; Yang, Z.; Wang, B. S.; Lv, K. Z.; Zhang, J. H. Effects of seaweed fertilizer on yield and agronomic characteristics of maize. Southwest China Journal of Agricultural Science, 2015,28(3):1166-1170. doi: 10.16213/j.cnki.scjas.2015.03.045 |
| 14 | Ran, M. L.; Song, G. H.; Ye, W. H. Effect of seaweed fertilizer on yield and quality of purple sweet potato. Journal of Huizhou University, 2014,34(6):18-22. doi: 10.3969/j.issn.1671-5934.2014.06.004 |
| 15 | Zhao, X. F.; Li, J. L. Study on application of algae fertilizer on vegetable production. Journal of Anhui Agricultural Sciences. 2009,37(6):2610,2616. doi: 10.3969/j.issn.0517-6611.2009.06.115. |
| 16 | Liu, J. P.; Liu, Y. L.; Shao, Y. Q.; Li, Y. H.; Wang, X. K.; Xue, R.; Li, C. L. Effect of seaweed compound fertilizer on yield and nutrient absorption and utilization of summer maize. Journal of Henan Agricultural University, 2021,55(3):429-434. doi: 10.16445/j.cnki.1000-2340.20210318.002 |
| 17 | Che, Q. Q.; Qiu, L. N.; Wang, Y. Z. Regulating effect of seaweed Bio-fertilizer on vegetative growth and fruit quality of Yanfu 3 apple. Modern Agricultural Science and Technology, 2020(24):50-52,66. doi: 10.3969/j.issn.1007-5739.2020.24.021 |
| 18 | Zheng, S. S.; Wu, J. H.; Jiao, F.; Ye, X. W.; Nie, L.; Wei, Y. G. Preliminary studies of the effect on alginic acid fertilizer applied to soybean. Journal of Heilongjiang Bayi Agricultural University, 2005,17(3):26-28. doi: 10.3969/j.issn.1002-2090.2005.03.007 |
| 19 | Wang, H. B.; Zhang, B.; Tao, J. J.; Chang, F.; Du, M. G.; Wang, Y. L. Alginic acid compound fertilizer: effects on yield and nutrient absorption and utilization of summer maize. Journal of Agriculture, 2017,7(10):25-29. |
| 20 | Gao, Y.; Han, X. H.; Wang, H. P.; Qin, Y. M.; Song, X. C.; Zhang, Y. Effects of alginic acid compound fertilizer on yield and quality of maize. South China Agriculture, 2020,14(2):147-149. doi: 10.19415/j.cnki.1673-890x.2020.02.068 |
| 21 | Zhu, Y. C.; An, G. L.; Li, W. H.; Liu, J. P.; Sun, D. X. Effects of alginate water soluble fertilizer on growth and quality of water-melon. Journal of Fruit Science, 2020,37(12):1898-1906. doi: 10.13925/j.cnki.gsxb.20200333. |
| 22 | Chen, S. B.; Chu, C. R. Effects of trehalate leaf fertilizer on quality of Guanyu loquat. Modern Agricultural Science and Technology, 2011(24):130-130,132. doi: 10.3969/j.issn.1007-5739.2011.24.078 |
| 23 | Zhang, R.; Wang, Y. X.; Zhao, X. H.; Li, L.; Fu, X. L.; Gao, D. S. Effects of different seaweed fertilizer levels on soil fertility and fruit quality of ‘Feicheng’ peach. Plant Physiology Journal, 2016,52(12):1819-1828. doi: 10.13592/j.cnki.ppj.2016.0030 |
| 24 | Feng, G.; Wang, P.; Zhang, D. M.; Gao, Y.; Qin, Y. M. Study on the application of seaweed organic water-soluble fertilizer in Chinese rose. Agriculture of Jilin, 2018(20):48-49. doi: 10.14025/j.cnki.jlny.2018.20.026 |
| 25 | Wang, H. P.; CiWang. D. J.; Gao, Y.; Zhang, L.; Qin, Y. M. Effect of seaweed organic and inorganic compound fertilizer on yield and quality of highland barley. Seed Science & Technology, 2019,37(13):42-43,45. doi: 10.3969/j.issn.1005-2690.2019.13.020 |
| 26 | Sun, H. M.; Shi, R. Y. Application effect of Haiguangyuan 818 seaweed fertilizer on cotton. Shihezi Science and Technology, 2003(3):7-8. doi: 10.3969/j.issn.1008-0899.2003.03.003 |
| 27 | Deng, X. L.; Lan, Y. Y.; Zhao, H. F.; Li, T. B., Wen, X. L. Fertilizer effect evaluation of organic water-soluble fertilizer with alginic acid on three kinds of leafy vegetables. Horticulture & Seed, 2020,40(7):1-3,13. doi: 10.16530/j.cnki.cn21-1574/s.2020.07.001 |
| 28 | He, M. L.; Tian, X.; Jiang, J.; Wang, C. H. Effects of foliar fertilizer produced from kelp waste on the growth and quality of color pepper and water bamboo. Jiangsu Journal of Agricultural Sciences, 2020,36(3):675-680. doi: 10.3969/j.issn.1000-4440.2020.03.020 |
| 29 | Wang, Z. L.; Zhang, J. J.; Sun, Q.; Wang, R. Evaluation of the effects of different organic fertilizer drip irrigation treatments on the quality of wine grapes and wines based on principal component and clustering analysis. Soils and Fertilizers Sciences in China, 2019(5):104-111. doi: 10.11838/sfsc.1673-6257.18463 |
| 30 | Yang, J. Y.; Yang, X. Analysis on several new water soluble fertilizers' effect on grape's output, quality and ecnomic benefits. Northern Horticulture, 2012(7):156-158. |
| 31 | Wu, Z. Y. Application effect of golden color fertilizer on cotton. Rural Science & Technology, 2014(1):40. doi: 10.3969/j.issn.1002-6193.2014.01.024 |
| 32 | Semaiti, T., Maulmu, Y. Application effect of golden color seaweed fertilizer on Hami Jujube. Northwest Horticulture, 2017(2):56-57. doi: 10.3969/j.issn.1004-4183.2017.02.033 |
| 33 | Wan, H. S. Trial result of kelp fertilizer on Udo. Gansu Agricultural Science and Technology, 2004(10):42-43. doi: 10.3969/j.issn.1001-1463.2004.10.020 |
| 34 | Yang, X. T.; Luo, S. Z.; Wang, X. H. Study on the application effect of Meiqi seaweed fertilizer on vegetables. Shanghai Agricultural Science and Technology, 2006(3):141-142. doi: 10.3969/j.issn.1001-0106.2006.03.139 |
| 35 | Xu, X. J.; Zhao, Z. Q.; Lu, Y.; Sun, M. S.; Zheng, Q.; Liu, Q. F. Effect of "Meiqi", a fertilizer from seaweed on peanut. Shandong Agricultural Sciences, 2005(1):57-58. doi: 10.3969/j.issn.1001-4942.2005.01.021 |
| 36 | Xiao, Z. Q. Application effect of Meiqi natural seaweed fertilizer on soybean. Modernizing Agriculture, 2010(5):14. doi: 10.3969/j.issn.1001-0254.2010.05.016 |
| 37 | Ma, M. M.; Gu, M. F.; Li, B. Effects of spraying seaweed fertilizer on yield and quality of pakchoi. Vegetables, 2021(12):28-31. |
| 38 | Li, Y. J.; Zhang, X.; Lu, J. S.; Wang, Y. Q.; Yang, Y. P.; Sun, Q. T.; Jiang, X. L.; Li, S. P.; Zhang, F. X. Effects of foliar fertilizer spraying on fruit setting rate and fruit quality of sweet cherry. China Fruits, 2013(05):34-36. doi: 10.16626/j.cnki.issn1000-8047.2013.05.024 |
| 39 | Wang, L. C.; Liu, Z. Z. Experiment on the effect of Meidina plant nutrient solution applied to apple. Northwest Horticulture, 1998, (6):10. |
| 40 | Wang, Y. X.; Li, F. D.; Li, Y. J.; Zhang, F. X.; Sun, Q. T.; Zhang, X.; Tian, C. P. Effect of three kinds of foliar fertilizers on the fruit quality of late ripening nectarine ‘Fuxiu'. Journal of Northeast Agricultural Sciences, 2018,43(4):41-43. doi: 10.16423/j.cnki.1003-8701.2018.04.010 |
| 41 | Zhang, G. Y.; Zhu, D. P.; Liu, Y. D.; Niu, J. J.; Chen, Q. R.; Naila Ilyas; Wei, J. Y.; Li, Y. Q. Effects of biochar and seaweed fertilizers on tobacco growth, soil properties and bacterial wilt occurrence. Chinese Tobacco Science, 2019,40(5):15-22. doi: 10.13496/j.issn.1007-5119.2019.05.003 |
| 42 | Li, S. W.; Wang, F. Z.; Chen, B.; Xie, J. M.; Shi, J. C. Effects of biochar combined with seaweed fertilizer on the physicochemical characteristics of continuous tobacco planting soil and flue-cured tobacco growth. Journal of Henan Agricultural University, 2021,55(5):852-861. doi: 10.16445/j.cnki.1000-2340.20210804.001 |
| 43 | Xue, X. M.; Han, X. P.; Chen, R.; Wang, L. P.; Wang, J. Z. Effects of biological organic fertilizer on growth and soil microbial community structure of red Fuji tree. Soils and Fertilizers Sciences in China, 2020(6):163-173. doi: 10.11838/sfsc.1673-6257.19501 |
| 44 | Xue, X. M.; Nie, P. X.; Han, X. P.; Chen, R.; Wang, J. Z. Effects of bio-organic fertilizer on tree, leaf, yield and quality of red fuji apple in early fruiting stage. Tianjin Agricultural Sciences, 2018,24(11):51-53,65. doi: 10.3969/j.issn.1006-6500.2018.11.014 |
| 45 | Huang, J. C.; Peng, Z. P.; Tu, Y. T.; Wu, X. N.; Liang, Z. X.; Yang, L. X.; Lin, Z. J. Yield, nitrogen and phosphorus nutrient effects of alginate compound fertilizer on double-cropping rice. Chinese Journal of Tropical Crops, 2020,41(5):859-867. doi: 10.3969/j.issn.1000-2561.2020.05.003 |
| 46 | Qu, C. X.; Wang, S. Y.; Xue, Y. Z.; Yin, C. Y.; Hu, X. M. Effect of application of new compound fertilizer "Beijing Leili seaweed fertilizer" on rice yield and components. Shanghai Agricultural Science and Technology, 2014(4):131-132. doi: 10.3969/j.issn.1001-0106.2014.04.083 |
| 47 | Tu, H. H.; Zhou, J.; Mao, Y.; Hu, X. X.; Kang, N. Q. Effect of natural seaweed fertilizer on plant growth and fruit quality of "Summer black" grape. Soils and Fertilizers Sciences in China, 2019(4):213-217. doi: 10.11838/sfsc.1673-6257.18336 |
| 48 | Wang, X. K.; Ma, J. Z.; Sun, Y.; Sun, J. K.; Yang, H. J.; Wang, X. J.; Wang, F. Q. Effect of a novel seaweed fertilizer on the growth and yield of maize. Journal of Yunnan Agricultural University (Natural Science), 2021,36(3):524-531. doi: 10.12101/j.issn.1004-390X(n).202006041 |
| 49 | Wang, X. K.; Wang, X. J.; Li, Feng.; Ma, N.; Xue, R. Research on application effect of new type phosphate fertilizer on maize. Phosphate & Compound Fertilizer, 2019,34(4):39-40. doi: 10.3969/j.issn.1007-6220.2019.04.016 |
| 50 | Xu, Q. X.; He, W. Z.; Zhang, J. New plant growth adjusting agent-research on utilization and effect of seaweed fertilizer on soybean. Soybean Science Technology, 2002(6):6-6. doi: 10.3969/j.issn.1674-3547.2002.06.006 |
| 51 | Sun, C. W.; Chen, Z.; Niu, S. K.; Zhao, Y. Z.; Wei, J. G.; Chu, F. J.; Yang, L. L. Effects of organic foliar fertilizers on growth physiology and fruit quality of Kyoho grape. Journal of Hebei Agricultural Sciences, 2016,20(5):28-30. doi: 10.16318/j.cnki.hbnykx.2016.05.008 |
| 52 | Sang, W. M.; Li, X. H. Application effects of efficient-enhancing compound fertilizers on potato. Shandong Agricultural Sciences, 2020,52(12):75-78. doi: 10.14083/j.issn.1001-4942.2020.12.014 |
| 53 | Zheng, H. X.; Xu, G. M.; Guan, A. N.; Feng, J. Effect of seaweed fertilizer on tomato yield and quality. Journal of Jilin Agricultural University, 1994, (S1):124-127. |
| 54 | Hao, X. L., Study on the effects of new organic fertilizers and biological agents on the control of potato soil-borne diseases. Journal of Inner Mongolia Agricultural University (Natural Science Edition), doi: 10.27229/d.cnki.gnmnu.2020.000105 |
| 55 | Wang, Z. L.; Bao, L.; Ge, X. W.; Wang, R.; Sun, Q. Effects of organic fertilization by drip irrigation on soil microbial biomass carbon,nitrogen and enzyme activities in wine vineyards. Soils and Fertilizers Sciences in China, 2019(2):61-67,97. doi: 10.11838/sfsc.1673-6257.18229 |
| 56 | Sun, X.; Yin, H. C.; Zhang, Z. T.; Song, W. L.; Yang, J. C.; Jiang, X. L.; Zhang, G. H.; Liu, Z. Y.; Ding, D. D.; Li, B. G. Effects of seaweed extract on yield and nutrient utilization of rice. Jiangsu Agricultural Sciences, 2020,48(16):100-103. doi: 10.15889/j.issn.1002-1302.2020.16.018 |
| 57 | Chen, W. D.; Zhou, W. L.; Ao, J. H.; Huang, Y.; Jiang, Y.; Han, X. H.; Qin, Y. M.; Shen, H. Effects of seaweed extract on yield, quality and nitrogen use efficiency of sweet corn. Crops, 2020(2):134-139. doi: 10.16035/j.issn.1001-7283.2020.02.020 |
| 58 | Xu, G. Y.; Yang, F. H.; Sun, F. F. Effects of water-soluble fertilizer containing seaweed extract on economic characters of potato. Yunnan Chemical Technology, 2021,48(4):47-49,61. doi: 10.3969/j.issn.1004-275X.2021.04.14 |
| 59 | Yu, H. L.; Xu, B. B.; Xu, G. Y.; Shao, W.; Qiao, X. S.; Si, P. Effect of seaweed extract combined with nutrients on the quality and nutrient absorption of grape fruit. Soils and Fertilizers Sciences in China, 2021(5):232-238. doi: 10.11838/sfsc.1673-6257.20371 |
| 60 | Wu, Y. P.; Wu, G. B.; Li, S. B.; Ye, Q. R.; Wei, X. Y. Effect of liquid seaweed manure on vegetable yield and quality. Northern Horticulture, 2006(5):16-18. doi: 10.3969/j.issn.1001-0009.2006.05.009 |
| 61 | Wang, Y. F.; Fu, F. Y.; Li, J. J.; Wang, G. S.; Wu, M. M.; Zhan, J.; Chen, X. S.; Mao, Z. Q. Effects of seaweed fertilizer on the growth of Malus hupehensis Rehd. seedlings, soil enzyme activities and fungal communities under replant condition. European Journal of Soil Biology, 2016,751-7. doi: 10.1016/j.ejsobi.2016.04.003 |
| 62 | Feng, C. S.; Liang, Z. Y.; Han, K. K.; Du, H. Q.; Lou, Z. G.; Liu, L. Effect of spraying seaweed fertilizer on yield and quality of alfalfa in returning green stage. Feed Research, 2023,46(20):133-136. doi:10.13557/j.cnki.issn1002-2813.2023.20.026 |
| 63 | Yu, H. L.; Xie, N.; Xu, G. Y.; Shao, W.; Si, P. Effects of seaweed compound water-soluble fertilizer on apple growth and fruit quality. Northern Horticulture, 2023(2):37-42. doi:10.11937/bfyy.20222059 |
| 64 | Xu, S. F.; Han, X. S.; Yang, K.; Chen, Y.; Tang, J. H.; Yu, X. M. Effect of seaweed organic water-soluble fertilizer on facility cherry tomato growth. Shanghai Vegetables, 2023(5):44-46. doi:10.3969/j.issn.1002-1469.2023.05.016 |
| 65 | Yu, H. L.; Xie, N.; Xu, G. Y.; Shao, W.; Xu, B. B.; Qiao, X. S.; Si, P. Effects of chemical fertilizer reduction combined with seaweed complex application on yield,fruit quality and nutrient absorption of grape. Journal of Fruit Science, 2022,39(4):584-592. doi:10.13925/j.cnki.gsxb.20210434 |
| 66 | Yang, F. L.; Liu, X. Y.; Yao, G.; Liu, H.; Li, H. Y. Experimental study on the efficiency of combining seaweed fertilizer and chemical fertilizer in the whole process of apple application. Journal of Fruit Resources, 2023,4(5):40-44. doi:10.16010/j.cnki.14-1127/s.2023.05.026 |
| 67 | Zhang, X.; Hu, X. H.; Hu, Z. P.; Liu, H.; Wang, Y. M.; Zhao, F. B.; Cheng, C. X.; Li, G. Z.; Zhu, D. P.; Yin, S. J.; Cheng, Y. D.; You, X. W.; Li, Y. Q. Effects of seaweed fertilizer and soybean seaweed fertilizer on flue-cured tobacco growth and soil microorganisms. Jiangsu Agricultural Sciences, 2023,51(3):81-88. doi:10.15889/j.issn.1002-1302.2023.03.012 |
| 68 | Li, Z. J.; Yu, L. T. Preliminary test report of organic water-soluble fertilizer on yield and quality of Yinghong No. 9 spring tea. Guangdong Tea Industry, 2022(4):12-14. doi:10.3969/j.issn.1672-7398.2022.04.003 |
| 69 | Zhou, M.; Yue, Z. F.; Yang, S. Q.; Zhang, Y.; Li, C.; Wang, S. M. Effect of application of seaweed fertilizer and water soluble inorganic fertilizer on growth and leaf nutrient of rubber small polytube-raised buddings. Chinese Journal of Tropical Agriculture, 2022,42(9):1-5. doi:10.12008/j.issn.1009-2196.2022.09.001 |
| 70 | Chen, J.; Liu, B.; Lei, W. W.; Chen, X. H.; Fan, S. S.; Chen, S. X.; Gao, F. Study on application effect of new fertilizer for greenhouse cucumber. Chinese Agricultural Science Bulletin, 2023,39(31):50-54. doi: 10.11924/j.issn.1000-6850.casb2022-0946 |
| 71 | Chen D , Zhou W , Yang J ,et al.Effects of Seaweed Extracts on the Growth, Physiological Activity, Cane Yield and Sucrose Content of Sugarcane in China.[J].Frontiers in plant science, 2021, 12:659130.DOI:10.3389/fpls.2021.659130. |
| 72 | Yao Y, Wang X, Chen B, Zhang M, Ma J. Seaweed Extract Improved Yields, Leaf Photosynthesis, Ripening Time, and Net Returns of Tomato (*Solanum lycopersicum* Mill.). ACS Omega. 2020 Feb 21;5(8):4242-4249. doi: 10.1021/acsomega.9b04155. |
| 73 | Meng L , Huang T , Shi J ,et al.Decreasing cadmium uptake of rice (*Oryza sativa* L.) in the cadmium-contaminated paddy field through different cultivars coupling with appropriate soil amendments[J].Journal of soil & sediments, 2019, 19(4):1788-1798.DOI:10.1007/s11368-018-2186-x. |
