# Supplementary material for: Effects of seaweed fertilizer application on crops’ yield and quality in field conditions in China-A meta-analysis
Source: PLoS One. 2024 Jul 18;19(7):e0307517. doi: 10.1371/journal.pone.0307517 (PMC11257332; doi:10.1371/journal.pone.0307517)
Supplement: S2 Table — (DOCX) [file pone.0307517.s002.docx]

S2 Table Group data heterogeneity of different effects of seaweed fertilizer with fixed model in meta-analysis

| **Seaweed fertilizer effects** | ***Q*** | **df** | ***P(*χ^2^)** | ***I*^2^** |
| --- | --- | --- | --- | --- |
| **Yield increasing** | 466.96 | 71 | 0.00 | 84.80 |
| **Growth improvement** | 2159.36 | 106 | 0.00 | 95.09 |
| **Quality improvement** | 2151.14 | 97 | 0.00 | 95.49 |

*Q*, the statistic value of heterogeneity for explaining variance; df, the degree of freedom; *P*(χ2), the

significant value of heterogeneity for explaining variance; *I*^2^, the variation in ln*R* attributable to heterogeneity.
